# A novel dominant *RPE65*-related retinopathy is caused by p.(E519K), a founder variant of Flemish origin

**DOI:** 10.21203/rs.3.rs-5849564/v1

**Published:** 2025-02-05

**Authors:** Eline Van Vooren, Filip Van den Broeck, Quinten Mahieu, Eline Geens, Mattias Van Heetvelde, Marieke De Bruyne, Stijn Van de Sompele, Sheetal Uppal, Eugenia Poliakov, Claire-Marie Dhaenens, Cheryl Y. Gregory-Evans, Lies Hoefsloot, Adriana Iglesias Gonzalez, Susanne Kohl, Theresia Zuleger, Tanguy Demaret, Dominant RPE65-p.(E519K) Consortium, Sari Tuupanen, Joke Ruys, Luc Van Os, Elise Platteau, Julie Jacob, Sascha Vermeer, Laurence Postelmans, Karin Dahan, Isabelle Maystadt, Florence Rasquin, Alberta A.H.J. Thiadens, Kirk A.J. Stephenson, Narin Sheri, Vasily Smirnov, Ian M. MacDonald, Kevin Gregory-Evans, T. Michael Redmond, Julie De Zaeytijd, Bart P. Leroy, Miriam Bauwens, Elfride De Baere

**Affiliations:** 1. Department of Biomolecular Medicine, Ghent University, Ghent, Belgium.; 2. Center for Medical Genetics, Ghent University Hospital, Ghent, Belgium.; 3. Department of Head and Skin, Ghent University, Ghent, Belgium.; 4. Department of Ophthalmology, Ghent University Hospital, Ghent, Belgium.; 5. Laboratory of Retinal Cell and Molecular Biology, National Eye Institute, NIH, Bethesda, United States.; 6. Univ. Lille, Inserm, CHU Lille, U1172-LilNCog-Lille Neuroscience & Cognition, F-59000 Lille, France.; 7. University of British Columbia, Department of Ophthalmology & Visual Sciences, Vancouver, BC, Canada.; 8. Department of Clinical Genetics, Erasmus Medical Centre, Rotterdam, The Netherlands.; 9. Institute for Ophthalmic Research, Centre for Ophthalmology, University Hospital Tübingen, Germany.; 10. Institute of Medical Genetics and Applied Genomics, University Hospital Tübingen, Germany.; 11. Centre de Génétique Humaine, Institut de Pathologie et de Génétique, Gosselies, Belgium.; 12. Blueprint Genetics, Espoo, Finland.; 13. Department of Ophthalmology, Vitaz, Sint-Niklaas, Belgium.; 14. Department of Ophthalmology, Antwerp University Hospital, Antwerp, Belgium.; 15. Department Ophthalmology, Maria Middelares Hospital, Ghent, Belgium.; 16. Department of Ophthalmology, UZ Leuven, Leuven, Belgium.; 17. Center for Human Genetics, UZ Leuven, Leuven, Belgium.; 18. Department of Ophthalmology, CHU Brugmann, Brussels, Belgium.; 19. Department of ophthalmology, Erasme Hospital, Université Libre de Bruxelles, Brussels, Belgium.; 20. Department of Ophthalmology, Erasmus Medical Centre, Rotterdam, The Netherlands.; 21. Department of Ophthalmology and Visual Sciences, University of Alberta, Edmonton, Canada.; 22. Sorbonne Université, INSERM, CNRS, Institut de la Vision, Paris, France.; 23. Division of Ophthalmology and Center for Cellular and Molecular Therapeutics, Children's Hospital of Philadelphia, Philadelphia, PA, United States.

**Keywords:** RPE65, founder variant p.(E519K), dominant late-onset macular dystrophy, molecular consequences, target for therapy

## Abstract

**Background ∣:**

Recessive *RPE65*-retinopathy is an inherited retinal disease (IRD) that is a well-known target for gene therapy. Dominant *RPE65*-retinopathy, however, due to the Irish founder variant p.(D477G), is very rare. Here, we present the discovery of a novel dominant *RPE65*-retinopathy caused by ultrarare variant c.1555G>A, p.(Glu519Lys), hereafter named p.(E519K).

**Methods ∣:**

In addition to genome (n=3), exome (n=28), or targeted sequencing (index: n=14; segregation: n=30) to identify the p.(E519K) variant, patients underwent extensive ophthalmological examinations. Haplotype phasing was based on long-read genome sequencing data in four individuals, combined with microsatellite analysis of six markers in all index cases. The p.(E519K) variant was functionally assessed using an enzymatic RPE65 assay, western blotting, co-immunoprecipitation, cellular thermal shift assay (CETSA), minigene assays and protein modelling (AlphaFold).

**Results ∣:**

Using genome, exome, or targeted sequencing in Belgian IRD cases (discovery cohort, n=2,873) and interrogating genomic IRD databases from France, the Netherlands, Germany, United Kingdom, Scotland, Ireland, and Canada (replication cohort, n=18,798) we identified 83 monoallelic p.(E519K)-IRD cases of Flemish ancestry. Long-read sequencing-based haplotyping revealed a shared region of 464 kb, confirming a founder effect. p.(E519K) affects a highly conserved amino acid and lowers RPE65 enzymatic activity to ~56%, in line with reduced protein expression. While no increased interaction with wild type RPE65 or aberrant splicing could be demonstrated *in vitro*, protein modelling and CETSA supports a shift in protein stability. Segregation analysis revealed dominant inheritance with complete penetrance and phenotypic variability, hallmarked by a characteristic late-onset macula-predominant IRD with two main subtypes. The milder phenotype is characterized by subtle, diffuse mottling of the posterior pole, while the more severe phenotype manifests as a macular pattern dystrophy with chorioretinal atrophy as a hallmark.

**Conclusions ∣:**

The discovery of a dominant *RPE65*-IRD due to the ultrarare Flemish founder variant p.(E519K) reduces the diagnostic gap in dominant IRD and highlights a novel target for therapy.

## Background

Approximately two million people worldwide are affected by inherited retinal disease (IRD), a clinically and genetically heterogeneous group of disorders leading to progressive vision loss or stable congenital visual impairment^1,2^. Over 290 genes have been implicated in IRD, one of which is *RPE65* (retinal pigment epithelium-specific 65 kDa), encoding a key enzyme in the visual cycle, which catalyzes the crucial isomerization of all-*trans* retinyl ester to 11-*cis* retinol^3–5^. Biallelic pathogenic variants in the *RPE65* gene (OMIM*180069) are implicated in different forms of autosomal recessive (AR) IRD, including Leber congenital amaurosis type 2, early-onset severe retinal dystrophy, and retinitis pigmentosa. Patients with AR *RPE65*-IRD manifest a generalized phenotype with common clinical findings including congenital or early-onset night blindness, progressive loss of visual field and loss of central vision^6^. Overall, AR *RPE65*-IRD represents 0.8-1.5% of all IRD cases, affecting around 15,620 patients worldwide^7–11^. In total, 359 and 800 *RPE65* variants, distributed across the entire gene, have been deposited in the Leiden Open Variation Database^12^ and ClinVar^13^ respectively, 281 and 230 of which are classified as (likely) pathogenic.

*Voretigene neparvovec-rzyl* (Luxturna^®^), the first gene therapy for IRD approved by FDA and EMA in 2017 and 2018 respectively, is a treatment for AR *RPE65*-IRD. This gene augmentation therapy involves subretinal injection of adeno-associated virus type 2 vectors containing wild type (WT) *RPE65* complementary DNA (cDNA), resulting in improved light sensitivity, visual field and navigational abilities^14–17^. Eligibility depends on the presence of biallelic (likely) pathogenic *RPE65* variants and sufficient viable outer retinal cells, emphasizing the importance of an early molecular diagnosis.

Autosomal dominant (AD) *RPE65*-IRD has been described only once, due to the Irish founder variant c.1430A>G p.(Asp477Gly), also known as p.(D477G). Only a few families have been identified worldwide, showing non-penetrance and large phenotypic variability, ranging from foveal vitelliform lesions in otherwise normal appearing retinae to extensive chorioretinal atrophy mimicking choroideremia^18–21^. Recapitulation of the p.(D477G)-IRD in knock-in mouse models was not apparent, with only minimal phenotypic manifestations observed^22–24^. Possible mechanisms of action involve aberrant RNA splicing and a toxic and/or dominant-negative effect through abnormal aggregate formation^22,24^.

Here, we describe the discovery, replication and molecular, functional, and clinical characterization of a novel AD *RPE65*-related retinopathy due to an ultrarare variant c.1555G>A, p.(Glu519Lys), hereafter referred to as p.(E519K). Our findings reduce the diagnostic gap in dominant IRD and highlight a novel target for therapy.

## Methods

### Patients

Index patients and (affected) family members were recruited from Belgian ophthalmology clinics and centers for medical genetics. The study was conducted in accordance with the principles of the Declaration of Helsinki and was approved by the Ethics Committee of Ghent University Hospital. All included patients consented to the study.

### Clinical investigations

The in-house p.(E519K) cohort, for which clinical investigations are described, consists of 65 patients, who have been examined at least once at the Department of Ophthalmology at Ghent University Hospital, Ghent, Belgium, the national referral center for ophthalmic genetics. Prior clinical records were revisited for general and ophthalmologic history, and for ophthalmological data recorded at first and follow-up visits. Ophthalmological data included best-corrected visual acuity (BCVA), ISCEV-standard full-field flash electroretinography in 50% of patients and electro-oculography in five cases (Roland Consult, Brandenburg an der Havel, Germany), anterior segment and dilated fundus examination supplemented with multimodal fundus imaging including spectral-domain optical coherence tomography (OCT), long-wavelength (690 nm) reflectance and short-wavelength (588 nm) autofluorescence (SW-AF) imaging with confocal scanning-laser ophthalmoscopy (Spectralis, Heidelberg Engineering GmbH, Heidelberg, Germany), color fundus photography (Clarus 700, ZEISS, Jena, Germany; and/or TRC-IX50 and TRC-DX50, Topcon Corporation, Tokyo, Japan).

BCVAs were measured using logMAR charts and are expressed in decimal equivalents. Values above 1.00 were limited to 1.00 as testing was not consistently continued beyond this point. Off-chart values were equalized with decimal values as previously described^25^. Five eyes of three patients were excluded for clinical analysis due to comorbidity with a more severe IRD or retinal trauma bringing the total to 125 eyes of 63 cases for clinical analysis. Of these, an additional five eyes (three cases) had their symptomatology and BCVAs excluded due to a comorbidity expected to affect these variables (Table 1). These factors are the reason the total number of cases (denominators) are not the same for every ratio presented.

### Short-read whole genome sequencing (srWGS)

srWGS was performed for patients F5-III:2, F5-IV:3 and F8-II:4. Library preparation and sequencing were performed using the Illumina DNA polymerase chain reaction (PCR)-Free LibraryPrep kit and 150 base pairs (bp) paired-end sequencing (S4, 300 cycles, NovaSeq 6000, Illumina, CA, USA). Data were basecalled using bcl2fastq2 (v2.20)^26^ and demultiplexed with ngsutils^27^. Basecalled reads were subsequently aligned to GRCh38 using BWA-MEM (v0.7.17)^28^, after which coverage was assessed using mosdepth (v0.3.3)^29^. Variant calling for single nucleotide variants (SNVs) was done using HaplotypeCaller from the GATK (v3.3)^30^ toolkit and annotation was performed through Ensembl Variant Effect Predictor (v110.0)^31^ and dbNSFP (v4)^32^. Quality control was summarized in MultiQC reports (v1.12)^33^, including FastQC (v0.12.0)^34^, samtools and bcftools (both v1.18)^35^ data. Post-demultiplexing steps were performed within the bcbio toolkit^36^. Resulting SNV VCF files were analyzed using the in-house tool Seqplorer (https://seqplorer.cmgg.be/user/login/) for coding variants in the RetNet panel (v7, 324 genes) and the online platform Franklin (Genoox, https://franklin.genoox.com/) for coding and non-coding variants in the same panel. Structural variants (SVs) overlapping with this gene panel were assessed using Manta (v1.6.0)^37^ and Delly (v0.8.7)^38^. Variants were classified using American College of Medical Genetics and Association for Clinical Genomic Science guidelines with adaptations^39^. The strength of some criteria was altered under certain conditions as recommended in literature^39–43^.

### Whole exome sequencing (WES)

A subset of index patients underwent WES, revealing p.(E519K) (Table 1, Supplementary Table 1). In addition, targeted sequencing of in-house index patients (F12I:2, F13II:1, F14II:2, S08, S09, S11, S13, S14, S17 and S18) was supplemented with WES to exclude the presence of additional (likely) pathogenic variants in IRD genes. Library preparation and sequencing were performed using the SureSelectXT Human All Exon V6 or V7 (Agilent, CA, USA), KAPA HyperExome V1 (Roche) kit or Haloplex target enrichment System (Agilent Technologies Inc., CA, USA) and 150 bp paired-end sequencing (HiSeq 3000 or NovaSeq 6000, Illumina, CA, USA). Basecalling, demultiplexing, alignment, variant calling and annotation was performed as described for srWGS data, using a bed file to restrict the analysis to coding regions. Resulting SNVs were filtered using the in-house WES analysis tool Seqplorer for the customized RetNet panel (v7). Copy number variations in these genes were assessed using ExomeDepth (v1.1.16)^44^.

An NGS-based assay, containing 351 IRD genes, was performed for patients F17-II:1, F17-II:2, S31 and S32 using the Blueprint Genetics Retinal Dystrophy Panel^45^, while patients S29 and S30 underwent sequencing of the Inherited Retinal Disorders Panel by Invitae, containing 330 genes^46^.

Patient S33 underwent high-throughput sequencing of a panel of 226 IRD genes. Coding exons and their flanking intronic regions were captured using the Haloplex target enrichment System (Agilent Technologies Inc., CA, USA). DNA libraries were sequenced on a NovaSeq sequencer (Illumina). Data analysis was done using an in-house developed pipeline compiling the data obtained from Seqnext (JSI Medical System, Ettenheim, Germany) and GATK software. Copy number variants (CNVs) detection was performed by a quantitative analysis based on the amplicons read depth as previously described^47^.

The DNA of patient S34 was enriched using Agilent SureSelect DNA, SureSelect OneSeq 300kb CNV Backbone and Human All Exon V7 capture, followed by paired-end sequencing (Illumina). Data were demultiplexed with bcl2fastq Reads were mapped using BWA-MEM. Variant calling was performed by the GATK HaplotypeCaller, followed by filtering and annotation with Alissa Interpret software and classification with Alamut Visual. CNV detection was done via the BAM multiscale reference method using depth of coverage analysis and dynamical bins in NexusClinical, which was also used to filter and annotate the detected CNVs.

### Targeted sequencing and segregation analysis

A subset of index patients (Table 1, Supplementary table 1) that had previously tested negative for mitochondrial retinopathy, or for *ABCA4*, *BEST1* or *PRPH2* targeted testing, underwent targeted sequencing for p.(E519K). Fifty-one family members underwent segregation analysis for p.(E519K). PCRs were performed using Kapa2G Robust Master Mix (2x, Kapa Biosystems, MA, USA), followed by Sanger sequencing using the BrilliantDye^™^ kit (v3.1, NimaGen, Nijmegen, Netherlands) and run on an ABI3730XL DNA analyzer (Applied Biosystems, MA, USA). Primers used for PCR and sequencing are listed in Supplementary Table 2.

### Long-read whole genome sequencing (lrWGS)

lrWGS was executed for patients F5-III:2, F5-IV:3, F8-II:3; and F8-II:4. Library preparation was performed using the SQK-LSK114 ligation sequencing kit (Oxford Nanopore Technologies (ONT), United Kingdom), followed by sequencing on a PromethION 24 system (ONT), using an individual FLO-PRO114M (ONT) flow cell for each sample, for a maximum of 72 hours (h). Basecalling was done through Guppy (v.6.4.6, ONT) after which reads were filtered on a minimum length of 100 bases and a minimum average read quality score of 10 using NanoFilt (v2.6.0)^48^. Reads were trimmed and mapped to GRCh38.p14 via minimap2 (v2.24)^49^. SVs were called with SVIM (v1.4.2)^50^, Sniffels2 (v2.0.7)^51^, and CuteSV (v2.0.2)^52^ after which a consensus VCF was generated using Jasmine (v1.1.5)^53^. SNVs were called using Clair3 (v1.0.5)^54^ and phased using WhatsHap (v2.0)^55^. Coverage analysis was done for all alignment files using mosdepth (v0.3.3)^29^ and quality metrics were assessed through pycoQC (v2.5.2)^56^ and NanoPlot (v1.40.0)^48^.

### Microsatellite analysis

Microsatellite markers (Supplementary Table 3) were PCR amplified with 10x PCR buffer (Invitrogen, MA, USA), 50 mM MgCl_2_ (Invitrogen), 1 mM dNTPs (New England Biolabs (NEB), MA, USA), DMSO (Sigma-Aldrich, MO, USA), 5 u/μl Taq (Invitrogen), and 10 μM primer. One μl of product was mixed with 0.3 μl ROX-500 size standard (GeneScan, Freiburg im Breisgau, Germany) and 10 μl Hi-Di formamide (Applied Biosystems). PCR fragments were size fractionated on an ABI3730XL capillary sequencer and data were analyzed using GeneMapper software (v5, Applied Biosystems).

### Generation of expression vectors and site-directed mutagenesis

Human *RPE65* and *CRALBP* cDNAs, obtained from gBlocks (IDT, IA, USA), were subcloned into bicistronic pVitro2 expression vector (InvivoGen, CA, USA). An expression vector containing *RPE65* cDNA in the pcDNA3.4 backbone was obtained from GenScript (New Jersey, USA). The p.(D477G) and p.(E519K) variants were introduced in the *RPE65* sequence in both vectors, and HA- and MYC-tags were introduced into the *RPE65*-pcDNA3.4 vector, using the Q5 Site-Directed Mutagenesis Kit (NEB). Primers used to amplify the gBlocks, mutagenesis PCR and PCR to verify the mutagenesis are listed in Supplementary Table 4.

### Cell culture and transient transfection

HEK 293-T cells (ATCC, Virginia, USA) were maintained in Dulbecco’s Modified Eagle Medium (DMEM, Gibco, Thermo Fisher, MA, USA) supplemented with 10% fetal bovine serum (FBS, Greiner Bio-One, Kremsmünster, Austria) and 1% Penicillin-Streptomycin (Gibco) at 37 °C with 5% CO_2_. Adult Retinal Pigment Epithelium-19 (ARPE-19, ATCC) cells were maintained in DMEM-F12 (Gibco), supplemented with 10% FBS, 1% Penicillin-Streptomycin, 1% Non-essential amino acids (Gibco), 1% Glutamax supplement (Gibco) and 0.1% Amphotericin B (Gibco). Cells were seeded, 0.75 – 3 x 10^4^ cells (12-well plate) and 2.2 x 10^6^ cells (10 cm culture dish) and transfected using Lipofectamine 3000 transfection agent (Invitrogen) or TransIT-X2 (Mirus Bio, WI, USA) with 1 mg/ml – 1 μg/ml vector, depending on the experiment. Negative control samples were transfected without vector.

For the *in vitro* isomerase activity assay, pVitro2 expression plasmids containing either WT-RPE65 or p.(E519K)-RPE65 were prepared by using QIAprep Maxi kits (Qiagen, CA, USA). Next, 30 μg of pVitro2 plasmid (WT or mutant) were transfected into human Expi293 suspension cells (Thermo Fisher Scientific) using 40 μl of ExpiFectamine transfection reagent (Thermo Fisher Scientific). The cells were incubated on an orbital shaker at 37 °C and 8% CO_2_ for 16-18 h. At 18 h post transfection Expifectamine 293 Enhancer 1 (150 mL/flask) and Expifectamine 293 Enhancer 2 (1.5 mL per flask) were added. All-*trans* retinol was added 42 h post-initial transfection, at a final concentration of 2.5 μM. Finally, cells were harvested at 6 h after substrate addition (total 48 h transfection to harvest) for downstream retinoid analysis.

### Retinoid Extraction, Saponification, and Retinoid Analysis

All procedures were performed under red safelights. For analysis of WT and p.(E519K)-RPE65 activity in Expi293 cells, retinoids were extracted and saponified as described^57^ from cells harvested by centrifugation from 30 ml volumes of cultured transfected Expi293 cells. Isomeric retinols were analyzed on 5 *μ*m particle LiChrospher Si-60 (Alltech, IL, USA) normal phase columns (2 × 250 mm) on an Agilent 1100/1200 series HPLC system (Agilent Technologies, DE, USA), in hexane mobile phase containing ethyl acetate (11.2%):dioxane (2.0%):octanol (1.4%), following Landers & Olson, 1988^58^, as earlier modified^57^. Data were analyzed using ChemStation32 software (Agilent).

### Western blot

Proteins were isolated using radioimmunoprecipitation assay buffer (RIPA, 94%, Sigma-Aldrich), protease inhibitor (4%, Roche, Basel, Switzerland), phosphatase inhibitor 2 (1%, Sigma-Aldrich), and phosphatase inhibitor 3 (1%, Sigma-Aldrich). Assessment of protein yields was performed according to the Pierce BCA protein assay protocol (Thermo Fisher). Total protein (15-25 μg) was separated by electrophoresis on Nu-Page 4-12% Bis-Tris Gel (Invitrogen) under reducing conditions and transferred to a nitrocellulose membrane (Invitrogen). Membranes were blocked with 5% membrane blocking agent (Cytiva, MA, USA) and incubated with either anti-RPE65 antibody (1:600, 17939-1-AP, Proteintech Europe, Manchester, UK), anti-CRALBP (1:4000, ab1501, Abcam, Cambridge, UK), or anti-ß-tubulin (1:2500, ab6046, Abcam) 1.5 - 2 h at room temperature. Membranes were washed with 1x Tris-buffered saline with 0.5% Tween20 and incubated with secondary anti-rabbit or mouse antibody (1:2500, Cell Signaling Technology, MA, USA). Membranes were developed with SuperSignal West Dura Extended Duration Substrate (Thermo Fisher). Expression levels of CRALBP were used to evaluate transfection efficiency and to normalize RPE65 expression. Quantification of RPE65 and CRALBP expression was performed using the ImageLab software (v6.1, Bio-Rad, CA, USA).

### Cellular thermal shift assay (CETSA)

Ten million HEK 293-T cells were transfected (single or co-transfected) with 1 mg/ml WT-RPE65, p.(D477G) and/or p.(E519K) expression vector as described above. Forty-eight h after transfection, the cells were pelleted, dissolved in 1 ml 1x PBS (Gibco) and distributed over 12 tubes and spun down. Eighty μl of PBS was removed and each tube was incubated at a specific temperature within the following range 30 – 66.9 °C. Proteins were extracted by repeated freeze-thaw cycles using liquid nitrogen, followed by addition of 80 μl 1x PBS and ultracentrifugation (Optima TLX ultracentrifuge, Beckman Coulter, CA, USA). The supernatant was transferred, and protein concentrations were determined by the Pierce BCA protein assay kit (Thermo Fisher). Western blot was performed using anti-RPE65 antibody as described above.

### Minigene assays

To test the effect of *RPE65* variants p.(E519K) and p.(D477G) on splicing *in vitro* splice assays were used. A 3.4 kb genomic segment of *RPE65* spanning exons 11-14 was amplified from human genomic control DNA (Roche). The WT-*RPE65* PCR product was restricted and subsequently subcloned into the pSPL3_2096 vector, a derivative of the exon-trapping vector pSPL3 (Invitrogen-Life Technologies, Carlsbad, CA, USA), containing two HIV-TAT exons (TAT1, TAT2), flanking the insert. Mutant constructs were obtained using the Q5 Site-Directed Mutagenesis kit (NEB, MA, USA). Integrity of constructs was assessed using ONT long-read sequencing (Plasmidsaurus, Eugene, OR, USA). Constructs were transfected in HEK293T cells using Lipofectamine 3000 (Invitrogen). After 24 h, total RNA was extracted (Rneasy Mini Kit, Qiagen Benelux, Antwerp, Belgium) and used as input to obtain cDNA (iScript Select cDNA synthesis kit, Bio-Rad Laboratories). cDNA was PCR amplified with pSPL3 exon primers and separated via agarose gel (2% TBE) electrophoresis, followed by Sanger sequencing (Applied Biosystems), next generation sequencing (NGS, Miseq, Illumina) and ONT long-read sequencing (Eurofins Genomics, Germany). Primers used for cDNA synthesis and cDNA PCR are listed in Supplementary Table 5.

To calculate exon ratios NGS and ONT data was aligned against the corresponding construct sequence using STAR (v2.7)^59^ and minimap2 (v2.24)^49^ respectively. Coverage for each position of the construct was determined using samtools depth (v1.18)^35^, and mean coverage was calculated for exons included in the construct. Cumulative bar graphs for the relative coverage for each region were made with ggplot2 (v3.4.3) in RStudio (v2023.12.1+402).

### Co-immunoprecipitation (Co-IP)

Plasmids expressing HA-tagged WT-RPE65 and MYC-tagged p.(D477G) or p.(E519K)-RPE65 were either individually or co-transfected at an equal molar ratio in ARPE-19 cells, using TransIT-X2 (Mirus Bio, WI, USA) at 1 mg/ml and a 3:1 TransIT-X2/DNA ratio. At 48 h post transfection, total cellular proteins were extracted as described above. Co-IP was performed with protein A Dynabeads following manufacturer’s protocol (Invitrogen, MA, USA) using the anti-MYC (2 μg, ab9106, Abcam), the anti-HA (2 μg, ab9110, Abcam) and the anti-IGG antibodies (4 μg, ab2410, Abcam). Western blot was performed as mentioned earlier with the abovementioned anti-MYC (1:2000) and anti-HA (1:4000) antibodies.

### RPE65 protein crystal structure and modeling with AlphaFold

All monomer protein structures were modeled using the standalone version of AlphaFold (v2.0.0)^60^. WT RPE65 was modeled using a fasta file containing the UniProt sequence (https://www.uniprot.org/uniprotkb/Q16518/entry#sequences) as the only input for the ‘–fasta_paths’ flag. For missense isoforms modeling, this sequence was adapted to the predicted amino acid sequence resulting from each respective missense variant. Dimer structure predictions were modeled using AlphaFold (v2.3.1) with the ‘multimer’ model preset. Fasta files, used as the only input for the ‘–fasta_paths’ flag, contained only the two respective monomer structure sequences for each dimer structure prediction of interest. The ranked0 structures and crystal structures, 3FSN^61^ and 4RYX^62^ were visualized using UCSF ChimeraX (v1.7.1, RBVI with support of the National Institutes of Health, University of California, CA, USA). ChimeraX was also used to perform an electrostatic surface analysis.

### *In silico* predictions

*In silico* (splice) prediction scores were obtained for NM_000329.3: c.1430A>G (chr1: g.68431085T>C) and NM_000329.3: c.1555G>A (chr1:g.68429823C>T) using dbNSFP (v4.7)^63,64^ , PolyPhen-2^65^, MutationTaster2021^66^, Mutscore^67^, AlphaMissense^68^, SpliceAI lookup (Max distance 5000)^69^ and Aggrescan^70^.

### Statistical analysis

Data visualization and statistical analyses were performed with GraphPad Prism v10.2.0 for macOS (GraphPad Software, MA, USA). A Kruskal-Wallis test with Dunn’s multiple comparisons test was used to compare protein expression of Western blot, while a non-linear regression was used to generate the melting curves for CETSA.

## Results

### A novel *RPE65*-related dominant retinopathy caused by monoallelic variant p.(E519K)

**Figure 1:**
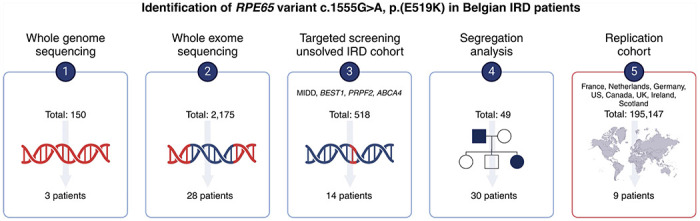


Short-read whole-genome sequencing was performed in three molecularly unresolved AD IRD patients (F5-III:2, F5-IV:3 and F8-II:4), which were prescreened via whole-exome sequencing following deep phenotyping for an IRD with predominant macular involvement. Prioritization of variants in known IRD genes revealed no clear-cut (non-)coding molecular diagnosis but uncovered instead an ultrarare, shared, monoallelic *RPE65* variant (NM_000329.3, exon 14), GRCh38/hg38 chr1:g.68429823C>T, c.1555G>A, p.(E519K), initially classified as a variant of unknown significance (VUS). The variant is present in gnomAD v2.1.1 (rs373274945) with an allele frequency of 0.0004014 (allele count: 1, no homozygotes). Revisiting the patients’ clinical records highlighted remarkable phenotypic similarities, clearly distinct from both the AR *RPE65*-IRD phenotype and the AD *RPE65*-IRD phenotype related to p.(D477G). Subsequently, in-house exome data from 2,175 individuals with IRD was queried, identifying 28 additional monoallelic p.(E519K) patients (Fig 1). As their phenotypes often resembled those seen in mitochondrial maculopathy or maculopathies associated with *ABCA4*, *BEST1*, and *PRPH2* variants, an IRD patient cohort that previously underwent targeted testing for these indications was screened, expanding the p.(E519K) cohort with 14 additional patients. The in-house index patients, identified using this targeted approach, underwent WES to exclude the presence of additional (likely) pathogenic variants in IRD genes. Subsequent segregation analysis revealed 30 clinically affected family members heterozygous for p.(E519K), confirming a dominant inheritance pattern we had initially established through pedigree analysis (Supplementary Fig 1). The genetic analyses performed as well as the presence of any additional variants, if applicable, are listed in Supplementary Table 1 for each IRD patient. In the initial IRD discovery cohort we found 75 monoallelic p.(E519K) IRD individuals that all are of Flemish origin (internal cases: n= 65; external referrals: n=10). Furthermore, we expanded our search to other IRD registries from those regions to which Flemish people migrated (France, the Netherlands, Germany, United Kingdom [UK], Scotland, Ireland, Canada), representing an IRD replication cohort of 18,796 individuals. Additional monoallelic p.(E519K) IRD cases were identified in the IRD registries from France (n=3, of which 1 (from Lille) included in this study), the Netherlands (Rotterdam, n=1), and Canada (British Columbia, n=4 and Alberta n=2). To obtain prevalence data in matched controls, we also mined non-IRD genomic databases from these regions (overview provided in Supplementary Table 6). Variant p.(E519K) was not found in any of the aforementioned non-IRD diagnostic genomic databases collectively representing 136,306 individuals (Supplementary Table 6).

In total, 85 monoallelic p.(E519K) individuals with IRD were identified in Belgium, France, the Netherlands and Canada. Genomic and clinical data of 83 out of 85 p.(E519K)-IRD patients are included in this study, of which 49 are from 17 families and 34 are sporadic patients.

### Index patients with p.(E519K) share a common haplotype of 464 kb

**Figure 2:**
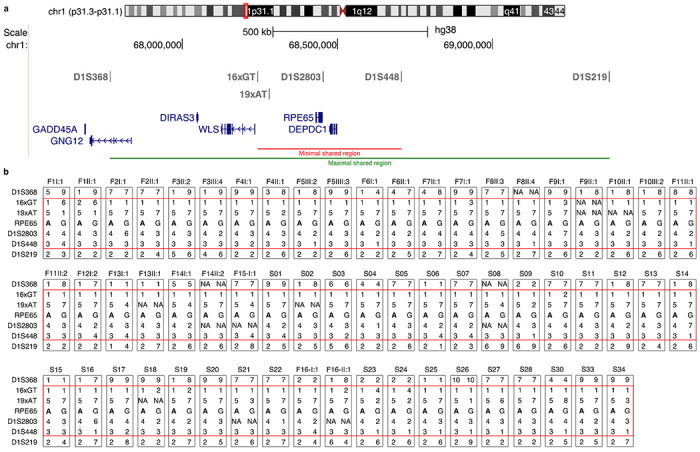


As the majority of IRD cases from the discovery cohort as well as from the replication cohort had a reported Flemish origin, a founder effect was suspected. Initial delineation of a common haplotype was performed using long-read WGS on four patients (F5-III:2, F5-IV:3, F8-II:3 and F8-II:4). Established boundaries were used for further haplotype reconstruction using six microsatellite markers flanking *RPE65* in all Belgian (in-house and external) index patients, one of their affected family members (if available), and the French (S33), Dutch (S34) and Canadian (S30) index cases (Fig 2). A minimal shared haplotype of 464 kb between all affected cases was identified, spanning *RPE65* and *DEPDC1*, while the maximal shared region of 1.6 Mb, additionally contains *WLS*, *DIRAS3* and part of *GNG12*. None of these genes, except for *RPE65*, are associated with IRD. Apart from p.(E519K), no other plausible (likely) pathogenic variants were found in the shared region.

### *In silico* and AlphaFold protein modeling supports a destabilizing effect of p.(E519K)

**Figure 3:**
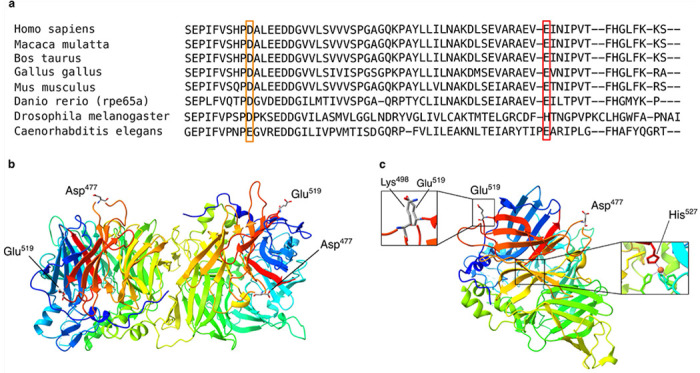


The RPE65 protein is evolutionarily well-conserved, which also applies to Glu^519^ and Asp^477^ (Fig 3a, Supplementary Fig 2a). When considering their paralogous residues through the wider carotenoid cleavage dioxygenase (CCD) superfamily, of which RPE65 is a member, Glu^519^ is conserved in all mammals, and in one of the three zebrafish ohnologs. In the frog and the other two zebrafish ohnologs, another carboxylic acid (aspartate) is present instead, also negatively charged and considered a Brønsted base, similar to glutamate. In the *Drosophila* CCD paralog, that position holds a histidine, also a Brønsted base (and acid).

To assess the potential pathogenicity of p.(E519K) and compare its properties with p.(D477G), 24 *in silico* predictions were evaluated. Overall, predictions for p.(E519K) compared to p.(D477G) were more damaging or deleterious (Supplementary Table 7). Moreover, Aggrescan predicts that both p.(D477G) and p.(E519K) are more prone to aggregation compared to WT RPE65 (Supplementary Fig 2b-d).

Furthermore, crystal structures of RPE65 were examined and AlphaFold models were generated to study the impact of p.(E519K) and p.(D477G) on protein structure and stability. The RPE65 protein exhibits a seven-bladed ß-propeller structure, with Glu^519^ situated on blade VII and Asp^477^ located on blade VI (Supplementary Fig 3). Both residues are positioned on the protein’s exterior, away from key sites such as the substrate tunnel, water tunnel and the dimerization site (Fig 3b). AlphaFold models for WT-RPE65, p.(E519K) and p.(D477G) show nearly identical folding, suggesting a minimal impact on protein structure (Supplementary Fig 4a-d). However, based on crystal structures of RPE65 (dimer: 3FSN and monomer: 4RYX), p.(E519K) may induce protein instability due to the proximity of a positively charged lysine (Lys^498^) causing a repulsion effect with Lys^519^ (Fig 3c and Supplementary Fig 4a). Moreover, Glu^519^ is located close to an important histidine residue (His^527^) on blade VII, crucial for iron binding in the catalytic center (Fig 3c and Supplementary Fig 3). Consequently, p.(E519K) could indirectly influence enzymatic RPE65 activity. In addition, the electrostatic surface analysis of both WT and p.(E519K) shows a shift from a negative potential, in the WT situation, to a positive potential at position 519 and the area around (Supplemental Figure 5). AlphaFold-Multimer was used to predict di- or multimer formation, revealing no differences in dimer formation compared to the crystal structure of the RPE65 dimer (Supplementary Fig 4e-j).

### Variant p.(E519K) affects expression, enzymatic activity and thermal stability of RPE65

**Figure 4:**
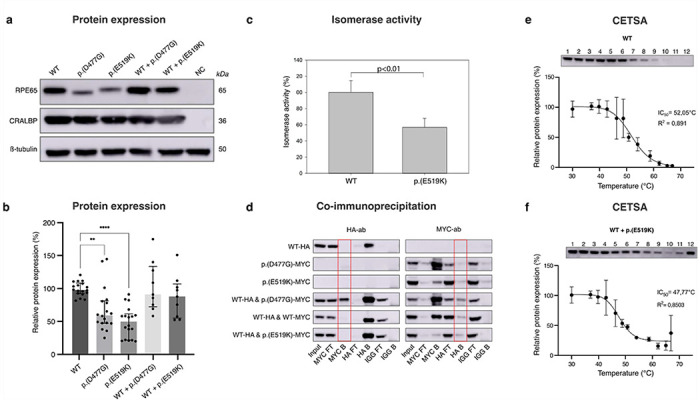


As p.(D477G) has been shown to alter splicing, minigene experiments in HEK293-T cells for both p.(D477G) (c.1430A>G) and p.(E519K) (c.1555G>A) were undertaken, although SpliceAI predictions only suggest a potential change for the former variant (Supplementary Fig 6a). Aberrant splicing was noted for p.(D477G), although in a less obvious manner than previously published, such as multiple exon skipping events with or without exon 13 skipping (Supplementary Fig 6b and c). Although usage of the strengthened cryptic acceptor site at c.1430 was noted for p.(D477G), this was not increased compared to WT or p.(E519K) (Supplementary Fig 6c)^23^. In our experiments, p.(E519K) does not seem to have an aberrant effect on splicing in HEK293-T cells, although a relatively increased ratio of reads containing exon 14 (incl. 3’UTR) was noted, possibly due to strengthening of the canonical acceptor site of exon 14 (Supplementary Fig 6d). Furthermore, overexpression studies were performed for p.(E519K) and p.(D477G) in HEK293-T and ARPE-19 cells. HEK293-T cells were single or co-transfected with WT-RPE65, p.(D477G) and/or p.(E519K) constructs, followed by immunoblotting. A reduced RPE65 protein expression of p.(D477G) and p.(E519K) compared to WT-RPE65 was found at 59.3% and 49.8%, respectively (p=0.0016 and p>0.0001), following single transfection (Fig 4a,b). Co-expression of WT/p.(D477G) and WT/p.(E519K) did not result in any significant reduction of protein expression (p>0.9999 and p=0.7605) (Fig 4a,b). Migration of the p.(E519K) mutant protein is similar to that of the WT protein, unlike p.(D477G), which migrates marginally faster, as previously shown^19^. In addition, we found that Expi293 cells transfected with the p.(E519K) mutant construct produced about 56% of the 11-*cis* retinol made by cells transfected with WT-RPE65 (p<0.01) (Fig 4c). A CETSA assay was used to assess the effect of p.(E519K) on thermal stability of RPE65. The inhibitory concentration (IC_50_) temperature, whereby 50% of the RPE65 protein is denatured and aggregated, showed a difference between WT, p.(D477G), p.(E519K), WT/p.(D477G) and WT/p.(E519K) (Fig 4e,f and Supplementary Fig 7). Mutant (co-) transfections resulted in an IC_50_ lower than WT (WT/p.(E519K) = 47.77°C; WT/p.(D477G) = 49.11°C; WT = 52.05°C), suggesting p.(D477G) and p.(E519K) missense mutations result in thermal instability. Interestingly, the WT/p.(E519K) condition showed a sudden increase of detected protein at 66.9 °C (Fig 4f).

Following overexpression experiments in ARPE-19 cells, co-immunoprecipitation was performed to evaluate the interaction between WT-RPE65 and p.(D477G) and p.(E519K) mutants. No (differential) interaction could be demonstrated between WT-RPE65 and p.(E519K), in contrast to p.(D477G), which clearly interacts with WT-RPE65 (Fig 4d).

### Deep phenotyping reveals two distinct and recognizable p.(E519K)-IRD phenotypes

Besides the identification of p.(E519K) in 63 molecularly unresolved in-house patients, the p.(E519K) variant has been found in two patients (F10-II:1 and S20) that were molecularly solved (biallelic *ZNF408* and *CEP290* pathogenic variants). As their genotype corresponds to a more severe early-onset disease, the p.(E519K)-IRD is masked (Supplementary Figures 8 and 9). For this reason, these patients were excluded from the clinical analyses. A uniform clinical characterization was performed in 63 in-house patients with AD p.(E519K)-IRD, 44 of whom are familial (F1-F15, Supplementary Fig 1). Thirty patients (50.0%) mainly complained of reduced visual acuity and central scotomata (median age at onset 57 years; range 24-76 years) (Table 1 and Supplementary Table 8). The other 30 (50.0%) did not report symptoms but showed clinical signs upon investigation following diagnosis of an affected family member (median age at diagnosis 53 years; range 18-74 years) (Table 1 and Supplementary Table 8). BCVA at last exam ranged from 0.01 to 1.00 (median 0.65, interquartile range (IQR) 0.55) in the symptomatic group, and from 0.70 to 1.00 (median 1.00, IQR 0.10) in the asymptomatic group (Supplementary Table 8). Full-field electroretinography, performed in 30 (47.6%), showed predominant rod-system involvement in 14 (46.7%) (Supplementary Table 8).

**Figure 5:**
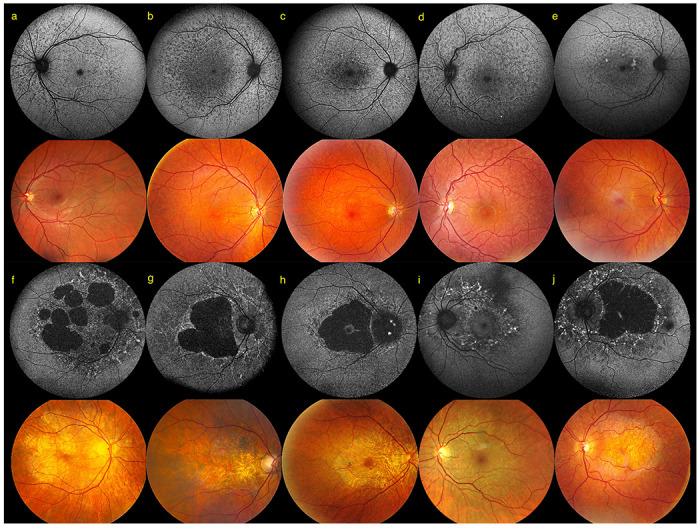


SW-AF imaging allowed categorization into two distinct phenotypes. A first phenotypic subgroup of 27 cases (43.5%) was referred to as a ‘Mottled Phenotype’ (Fig 5a–e) because of the presence of a collection of small, diffuse, hypo-autofluorescent (hypo-AF) spots spread over the posterior pole. These usually extended beyond the vascular arcades and the optic nerve head up to the retinal midperiphery. This hypo-AF mottling was often associated with variable hyperautofluorescent (hyper-AF) lesions. The second phenotype was classified as a ‘Pattern Dystrophy’ observed in a subgroup of 35 cases (56.5%) and is characterized by the presence of more organized hyper-AF changes as the predominant feature (Fig 5f–j).

Macular chorioretinal atrophic lesions were seen in 55 eyes (44.0%) and associated in all but one eye (S13), with the pattern dystrophy phenotype. Although patients with chorioretinal atrophy were often affected bilaterally (22; 68.8%) unilateral lesions were also noted (10; 31.3%) (Supplementary Table 8).

Color fundus images often showed rather subtle changes compared to SW-AF imaging and included diffuse depigmentation, fine white speckling and sometimes larger whitish to yellowish lesions which correspond to hyper-AF lesions (Fig 5). Vitelliform lesions were observed in 11 eyes (8.8%). These lesions were always unifocal and located at the fovea (Supplementary Fig 10). Electro-oculography was performed in four of these six cases with two cases having mildly subnormal light peak-to-dark trough ratios of 1.7-1.8. Cystoid macular edema was observed in 15 eyes (12.0%), seven of these had a concomitant vitelliform lesion. Structural retinal layer abnormalities were primarily located at the level of the three outermost hyperreflective bands on OCT. The innermost of these, the ellipsoid zone (EZ), often showed a fine sinusoidal deformation. More severe EZ alterations such as interruptions, granularity, and clumping were also observed, often in older eyes. Lesions hyper-AF on SW-AF imaging appeared as hyperreflective, tall, smooth, bell-shaped lesions or as wider-than-tall lesions with an irregular surface arising from the hyperreflective band corresponding to the RPE-Bruch membrane complex. Scans through chorioretinal atrophic lesions often showed degenerative intraretinal cavitation and outer retinal tubulations, which are both nonspecific signs often seen in retinal areas with profound outer retinal tissue loss (Supplementary Fig 11). The phenotypes observed in the patients that were referred by external IRD experts (Belgium, the Netherlands, France and Canada: S21-S32, F16-I:1, F16-II:1, F17-II:1, F17-II:2) are indistinguishable from those seen in the in-house IRD cohort (Supplementary Fig 12). One outlier is patient S21, displaying a retinitis pigmentosa-type rod-cone dystrophy (RCD). No other coding (likely) pathogenic variants could be identified using WES that could explain the RCD features (Supplementary Fig 13). Pathogenic variant(s) in non-coding regions, complex structural variants or variants in novel IRD genes cannot be excluded.

### Variant classification of c.1555G>A, p.(E519K)

The American College of Medical Genetics and Association for Clinical Genomic Science (ACMG/AMP) guidelines were used for the classification of the novel *RPE65* variant (Supplementary Table 10). The variant is present in population database gnomAD (v2.1.1) with an allele frequency of 0.00040114%, only one allele present, no homozygotes reported and the maximum population-specific frequence is 0.0008962% (PM2_PP). The described phenotype is specific for a disease with a single etiology (all associated genes have been analyzed, using WES or WGS, and variants in these genes are usually causal) after discussion with an experienced ophthalmologist (PP4_PM). In addition, the variant co-segregates with disease in multiple affected family members (31 family members; PP1_PS). Even with a conservative approach, excluding computational and functional data, the above-mentioned arguments result in a class 4 or a likely pathogenic variant based on the data described in this manuscript.

## Discussion

In this study, we present and characterize *RPE65* variant p.(E519K) as a novel and recurrent cause of dominant retinopathy in IRD cases of Flemish heritage, with the associated IRD often manifesting as an adult-onset macula-predominant retinal dystrophy.

The common haplotype of 464 kb, shared by all p.(E519K)-IRD patients of the discovery cohort, was also shared by the genotyped index cases of the replication cohort, supporting a founder effect. The absence of p.(E519K) in IRD patients originating from the Southern part of Belgium suggests a Flemish origin of p.(E519K). Hence, the presence of p.(E519K) in IRD patients from those regions where Flemish people migrated to (Flemish diaspora), specifically France, the Netherlands and Canada, is not surprising. Its overall rarity in gnomAD v2.1.1 as well as in the other genomic databases mined emphasizes the importance of increasing genetic ancestral diversity in population-scale datasets^71^. Indeed, this allows to interpret a larger number of rare variants^71^.

The properties of p.(E519K) were studied extensively by both *in silico* and *in vitro* analyses. Interestingly, another *RPE65* variant has been reported at this specific location, c.1555G>T, p.(Glu519Ter), in the context of AR *RPE65*-IRD, suggesting that this guanine is prone to variation^72^. *In silico* predictions and (dimer) protein modelling, using AlphaFold, support pathogenicity of p.(E519K) even more so than for p.(D477G). Protein instability of p.(E519K) can be explained by a repulsion effect between Lys^519^ and a neighbouring lysine (Lys^498^), the shift in surface potential, as well as a potential destabilising effect on a histidine residue (His^527^), essential for iron binding in the catalytic centre. Moreover, our *in vitro* experiments revealed a significantly reduced protein expression (49.8%) of p.(E519K) in HEK293-T cells. In addition, we demonstrate reduced isomerase enzymatic activity (56%) of p.(E519K) compared to WT when measured in an *in cellulo* minimal visual cycle assay. Both these values are comparable to those observed for p.(D477G)^23,24^. The level of RPE65 loss of function (LOF) observed here *in vitro* is highly unlikely to be the only disease mechanism, as carriers of recessive LOF *RPE65* variants exhibit no phenotype. However, cell type-specific effects, not captured *in vitro*, might contribute to a larger LOF effect. Moreover, both p.(D477G) and p.(E519K) induce thermal instability, as shown by CETSA. These results, together with an increased aggregation hot spot predicted by Aggrescan, support that p.(E519K) is prone to aggregation, a known pathogenic mechanism described for p.(D477G) and for variants in other IRD genes^24,73,74^. The interaction between p.(D477G) and WT-RPE65, experimentally demonstrated by Wu *et al.* ^24^, was confirmed using Co-IP in ARPE-19 cells. However, no interaction was observed between p.(E519K) and WT-RPE65, rendering a direct dominant negative effect mechanism in this cell type less likely. While aberrant splicing has been demonstrated for p.(D477G)^23^, our minigene results, performed in HEK293T cells, did not demonstrate a clear aberrant effect on splicing for p.(E519K), which is supported by its SpliceAI predictions. The impact of the observed result, a relatively increased ratio of reads containing exon 14 (including the 3’UTR), possibly due to strengthening of the canonical acceptor site of exon 14, is not clear in our *in vitro* set-up. Nevertheless, splicing could still be affected, in a more prominent and possibly different manner, in the RPE cells of the patients as splicing is highly species- and cell-type specific ^22–24,75–79^.

Several knock-in p.(D477G) mice (both WT/KI and KI/KI) have been generated to elucidate its pathomechanisms^22,23,76,77,80^. The resulting phenotypes were highly variable and dependent on age, luminescence exposure and genetic background. These mice lacked a discernible phenotype and thus did not accurately recapitulate the human situation. However, an p.(E519K) knock-in mouse model has been generated and will be studied to glean potentially useful parallels. The exact mechanism through which p.(E519K) (and, arguably, p.(D477G)) causes the dominant retinopathy thus remains elusive and more biologically relevant models, such as patient-derived induced pluripotent stem cell-derived retinal pigment epithelium (iPSC-RPE), are needed to mimic the native conditions of RPE65, including its native splicing, expression, localisation and interactions patterns.

**Figure 6:**
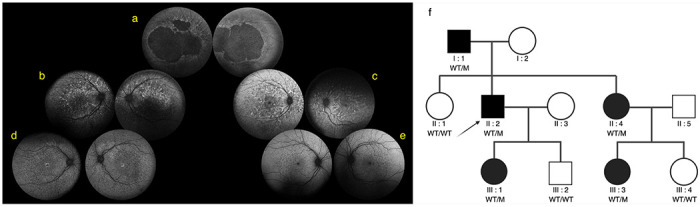


In addition to the molecular characterization of p.(E519K), a standard ophthalmological investigation was performed for all patients and an extensive clinical characterization was performed for the internal cohort of 63 Flemish patients. So far, only the p.(D477G)-IRD phenotype had been described and displayed reduced or non-penetrance. In contrast to the generalized, progressive, severe rod-cone dystrophy associated with AR *RPE65*-IRD^6,81,82^, p.(D477G)-IRD was initially described as an adult-onset choroideremia-like IRD^19^. Subsequently, it was described as displaying two clinical subtypes: one with foveal vitelliform deposits without peripheral degeneration^18^ and another with extensive chorioretinal atrophic lesions but with prominent early visual acuity disturbances and relative preservation of the anterior retina, distinguishing it from classic choroideremia^18,19^. In the Belgian p.(E519K)-IRD cohort, two distinct phenotypic subtypes were observed. The hypo-AF mottled subtype (43.5%) is characterized by a collection of small, diffuse, hypo-AF spots spread out across the posterior pole. This phenotype seems pathognomonic for this novel AD *RPE65*-IRD and could guide towards its molecular diagnosis. A second predominant phenotypic subtype (56.5%) displays a pattern dystrophy most often resembling the mitochondrial retinopathy associated with the *MT-TL1* variant m.3243A>G^83^. We hypothesize that the mottled phenotype might evolve into the more severe pattern dystrophy over time, with the two phenotypes representing the milder and more severe end of the p.(E519K)-IRD phenotypic spectrum, respectively. To support this, long-term follow-up of affected individuals will be required. Although an extensive variability for disease severity was noted in the p.(E519K) cohort (Fig 6), lower visual acuities and larger surface areas of chorioretinal atrophic lesions tended to be associated with older age. Based on segregation data and clinical investigations, we can conclude that p.(E519K)-IRD is fully penetrant. Indeed, all cases showed clinical signs upon retinal evaluation, albeit they were sometimes subtle and often not associated with visual symptoms. This raises the question whether the reported non-penetrance of p.(D477G)-IRD^18,19^, based on the absence of symptoms in a few obligate and confirmed carriers, could be revoked upon performing deep phenotyping in these cases.

Several diseases can be considered in the differential diagnosis of the p.(E519K) pattern dystrophy phenotype. These include mitochondrial retinopathies as well as *ABCA4*-IRD and *PRPH2*-IRD. Multiple atrophic lesions in patients above 50 years could be misdiagnosed as geographic atrophy in dry age-related macular degeneration (AMD). However, the additional presence of a pattern dystrophy on SW-AF imaging is different from AMD. For those patients in whom vitelliform lesions are predominant, other AD IRD caused by monoallelic variants in *BEST1*, *IMPG1*, *IMPG2* and *PRPH2* need to be considered.

Overall, our findings are of particular relevance in light of the approved *RPE65* gene augmentation therapy *Voretigene neparvovec-rzyl* (Luxturna^®^) for patients with AR *RPE65*-IRD. Unravelling the pathogenic mechanisms by which p.(E519K) and p.(D477G) cause AD *RPE65*-IRD, is thus of utmost importance in view of potential treatment options. For AD p.(D477G)-IRD several therapeutic attempts have been conducted: gene augmentation therapy has been performed in a p.(D477G) knock-in murine model, resulting in improvement of retinal function^80^. Moreover, oral 9-*cis* retinyl acetate therapy has been administered to five p.(D477G) patients, three of whom displayed improved visual function^80,84^. However, the long-term effects of either of these approaches on mutant toxicity and retinal degeneration are not yet known. Gene augmentation therapy would only prove beneficial when the mechanism of action includes LOF including haploinsufficiency. As mentioned above, these mechanisms alone are unlikely to cause the phenotype of AD *RPE65*-IRD as carriers of recessive LOF variants show no clinical signs. A gain-of-function or dominant negative effect, possibly combined with a LOF seem more plausible pathomechanisms and in these scenarios, augmentation therapy could induce toxic effects^85,86^. A potential therapy could involve antisense oligonucleotides (ASOs) to specifically target the mutant allele, which could be degraded. Proof-of-principle studies using ASOs have been performed for several IRD genes, including *RHO*^87^, *USH2A*^88^ and *CEP290*^89,90^, resulting in multiple follow-up studies and clinical trials (NCT03780257, NCT04123626, NCT03140969, NCT03913143 and NCT03140969)^91–95^. Gene editing, including CRISPR/Cas9-based, base or prime editing, is another therapeutic option for this variant. This would allow to permanently correct the mutation similarly to EDIT-101,the CRISPR/Cas9-based therapy targeting the *CEP290* IV26 variant (NCT03872479)^96,97^. As a gene therapy approach is dictated by the mechanism of a dominant mutation, insight into AD IRD pathogenesis is mandatory.

Comprehensive genetic testing is the key to establishing a definite and early diagnosis in suspected p.(E519K)-IRD cases, which is already crucial to provide adequate counselling and will only gain importance as therapies for this and similar retinal disorders will become available.

## Conclusions

Our study provides strong genetic, molecular, functional, and clinical evidence for a novel, adult-onset dominant *RPE65*-associated retinopathy due to a Flemish founder variant p.(E519K). These findings substantially expand the molecular and clinical spectrum of *RPE65*-IRD, reduce the diagnostic gap in dominant IRD, and open potential new therapeutic perspectives for AD *RPE65*-IRD.

## Supplementary Material

This is a list of supplementary files associated with this preprint. Click to download.

• SupplementarytablesandfiguresADRPE65.pdf

## Figures and Tables

**Table 1.** Demographics, Phenotype, Genetic testing method used to identify the c.1555G>A p.(E519K) RPE65 variant and optional remarks for each patient.

ID	Sex	Age at last exam (y)	Age at onset/diagnosis (y)	Phenotype	Genetic testing	Remarks
F1-I:1	M	61	61	Pattern Dystrophy	Targeted sequencing (segregation)	
F1-II:1	F	29	18	Mottled Phenotype	WES RetNet	
F2-I:1	M	90	90	Pattern Dystrophy	Targeted sequencing (segregation)	Left eye excluded due to trauma; right eye BCVA excluded due to cognitive problems
F2-II:1	F	63	63	Mottled Phenotype	Targeted sequencing (segregation)	
F2-III:1	F	34	18	Mottled Phenotype	WES RetNet	
F3-II:2	F	75	72	Pattern Dystrophy	WES RetNet	
F3-II:4	M	72	72	Pattern Dystrophy	Targeted sequencing (segregation)	
F3-III:4	F	48	47	Mottled Phenotype	Targeted sequencing (segregation)	
F3-III:6	F	45	45	Mottled Phenotype	Targeted sequencing (segregation)	
F3-III:8	F	38	38	Mottled Phenotype	Targeted sequencing (segregation)	
F4-I:1	M	79	72	Pattern Dystrophy	WES RetNet	
F4-II:1	M	58	58	Pattern Dystrophy	Targeted sequencing (segregation)	
F4-III:1	F	18	18	Mottled Phenotype	Targeted sequencing (segregation)	
F5-III:1	F	75	74	Pattern Dystrophy	Targeted sequencing (segregation)	
F5-III:2	M	69	60	Pattern Dystrophy	WGS RetNet	
F5-III:4	F	70	65	Pattern Dystrophy	Targeted sequencing (segregation)	
F5-III:5	F	68	64	Pattern Dystrophy	Targeted sequencing (segregation)	
F5-III:6	F	67	63	Mottled Phenotype	Targeted sequencing (segregation)	
F5-III:7	F	62	58	Pattern Dystrophy	Targeted sequencing (segregation)	
F5-III:8	F	60	56	Mottled Phenotype	Targeted sequencing (segregation)	
F5-IV:3	F	41	41	Pattern Dystrophy	WGS RetNet	
F6-I:1	M	68	33	Mottled Phenotype	Targeted sequencing (segregation)	
F6-II:1	F	41	29	Mottled Phenotype	WES RetNet	
F7-I:1	M	65	62	Mottled Phenotype	Targeted sequencing (segregation)	
F7-II:1	F	40	36	Mottled Phenotype	WES RetNet	
F8-II:3	M	65	58	Pattern Dystrophy	Targeted sequencing (segregation)	
F8-II:5	M	62	45	Pattern Dystrophy	WGS RetNet	
F8-III:1	F	40	40	Mottled Phenotype	Targeted sequencing (segregation)	
F8-III:2	F	37	37	Mottled Phenotype	Targeted sequencing (segregation)	
F9-I:1	M	72	72	Pattern Dystrophy	Targeted sequencing (segregation)	
F9-II:1	M	42	37	Mottled Phenotype	WES RetNet	
F10-II:1	M	68	NA	Autosomal recessive RCD	WES RetNet	IRD due to *ZNF408*: c.[16G>T] ; [16G>T], p.[ (Glu6Ter) ] ; [ (Glu6Ter) ]
F10-III:2	F	35	31	Mottled Phenotype	Targeted sequencing (segregation)	
F11-II:1	F	53	48	Mottled Phenotype	WES RetNet	Compound heterozygous carrier of two *ABCC6* variants without PXE phenotype
F11-II:2	F	46	39	Mottled Phenotype	Targeted sequencing (segregation)	BCVA excluded due to history of bilateral optic neuritis
F12-I:2	F	72	71	Pattern Dystrophy	Targeted sequencing (*PRPH2* negative cohort)	
F13-I:1	F	70	70	Mottled Phenotype	Targeted sequencing (segregation)	
F13-II:1	F	31	31	Mottled Phenotype	Targeted sequencing (specific phenotype)	
F14-I:1	M	86	70	Pattern Dystrophy	Targeted sequencing (segregation)	
F14-II:2	M	59	31	Pattern Dystrophy	Targeted sequencing (*PRPH2* negative cohort)	
F14-II:4	F	55	48	Pattern Dystrophy	Targeted sequencing (segregation)	
F14-III:2	F	30	30	Mottled Phenotype	Targeted sequencing (segregation)	
F14-III:3	F	27	27	Mottled Phenotype	Targeted sequencing (segregation)	
F15-I:1	M	81	75	Pattern Dystrophy	WES RetNet	
F15-II:1	M	57	57	Pattern Dystrophy	Targeted sequencing (segregation)	
S01	M	44	24	Mottled Phenotype	WES RetNet	
S02	M	44	35	Mottled Phenotype	WES RetNet	
S03	F	49	49	Mottled Phenotype	WES RetNet	
S04	F	51	50	Pattern Dystrophy	WES RetNet	
S05	M	54	49	Pattern Dystrophy	WES RetNet	
S06	M	57	55	Pattern Dystrophy	WES RetNet	
S07	M	57	57	Pattern Dystrophy	WES RetNet	
S08	F	59	59	Pattern Dystrophy	Targeted sequencing (*ABCA4* negative cohort)	
S09	F	59	54	Pattern Dystrophy	Targeted sequencing (MIDD negative cohort)	
S10	F	59	56	Pattern Dystrophy	WES RetNet	
S11	M	64	57	Pattern Dystrophy	Targeted sequencing (MIDD negative cohort)	
S12	F	66	59	Pattern Dystrophy	WES RetNet	
S13	M	67	57	Mottled Phenotype	Targeted sequencing (MIDD negative cohort)	BCVA excluded due to idiopathic congenital nystagmus
S14	M	70	70	Pattern Dystrophy	Targeted sequencing (MIDD negative cohort)	
S15	M	73	70	NA	WES RetNet	Short-wavelength autofluorescence imaging not performed
S16	M	77	76	Pattern Dystrophy	WES RetNet	Hemizygous carrier of *CACNA1F* variant without CSNB phenotype (normal ffERG)
S17	M	77	72	Pattern Dystrophy	Targeted sequencing (MIDD negative cohort)	
S18	M	78	74	Pattern Dystrophy	Targeted sequencing (MIDD negative cohort)	
S19	F	81	75	Pattern Dystrophy	WES RetNet	
S20	F	78	NA	Leber Congenital Amaurosis	WES RetNet	IRD due to *CEP290*: c.[4723A>T] ; [2991+1655A>G], p.[ (Lys1575Ter) ] ; [ ? ]
S21	F	55	55	RCD/RP	WES RetNet	External patient (Belgium) Toxic alcohol/tobacco-related optic neuropathy
S22	M	65	58	Mottled Phenotype	WES RetNet	External patient (Belgium)
F16-I:1	M	82	55	Pattern Dystrophy	Targeted sequencing (segregation)	External patient (Belgium)
F16-II:1	F	60	57	Pattern Dystrophy	WES RetNet	External patient (Belgium)
S23	M	58	48	Pattern Dystrophy	WES RetNet	External patient (Belgium)
S24	M	44	39	Mottled Phenotype	Targeted sequencing (*PRPH2* & *BEST1* negative cohort)	External patient (Belgium)
S25	F	65	65	Pattern Dystrophy	Targeted sequencing (*PRPH2* & *BEST1* negative cohort)	External patient (Belgium)
S26	M	70	63	Pattern Dystrophy	Targeted sequencing (MIDD negative cohort)	External patient (Belgium)
S27	M	69	55	Pattern Dystrophy	Targeted sequencing (MIDD negative cohort)	External patient (Belgium)
S28	M	40	37	Pattern Dystrophy	WES RetNet	External patient (Belgium)
F17-II:1	F	44	33	Mottled Phenotype	Retinal Dystrophy Panel (Blueprint Genetics)	External patient (Canada)
F17-II:2	F	40	35	Mottled Phenotype	Retinal Dystrophy Panel (Blueprint Genetics)	External patient (Canada)
S29	M	57	55	Mottled Phenotype	Inherited Retinal Disorders Panel (Invitae)	External patient (Canada)
S30	F	38	35	Mottled Phenotype	Inherited Retinal Disorders Panel (Invitae)	External patient (Canada)
S31	M	72	60–70	Pattern Dystrophy	Retinal Dystrophy Panel (Blueprint Genetics)	External patient (Canada)
S32	M	65	50–60	Pattern Dystrophy	Retinal Dystrophy Panel (Blueprint Genetics)	External patient (Canada)
S33	M	24	17	Mottled Phenotype	Targeted sequencing 226 IRD genes	External patient (France)
S34	M	73	NA	Pattern Dystrophy	WES Vision disorders (505 IRD genes)	External patient (Netherlands)

External patients were seen in Belgian clinical centers other than Department of Ophthalmology at Ghent University Hospital. Abbreviations used: M = male; F = female; RCD = rod cone dystrophy; WES = whole exome sequencing; WGS = whole genome sequencing; MIDD = Maternally Inherited Diabetes and Deafness; NA = not available/applicable; PXE = Pseudoxanthoma elasticum ; CSNB = Congenital Stationary Night Blindness ; ERG = Electroretinogram.

## Data Availability

Extended data generated in this study are available in the supplementary materials.

## Dominant RPE65-p.(E519K) Consortium

Lonneke Haer-Wigman, Susanne Roosing, Department of Human Genetics, Radboud University Medical Center, Nijmegen, The Netherlands.

Andrew R. Webster, Siying Lin, Gavin Arno, Omar Mahroo, National Institute of Health Research Biomedical Research Centre at Moorfields Eye Hospital and the UCL Institute of Ophthalmology, London, UK.

Roly Megaw, MRC Human Genetics Unit, MRC Institute of Genetics & Cancer, University of Edinburgh, Western General Hospital, Edinburgh, EH4 2XU, UK & Princess Alexandra Eye Pavilion, NHS Lothian, Chalmers St, Edinburgh, EH3 9HA, UK

Mihail Halachev, MRC Human Genetics Unit, MRC Institute of Genetics & Cancer, University of Edinburgh, Western General Hospital, Edinburgh, EH4 2XU, UK

Anne Lampe, South East of Scotland Clinical Genetics Service, Western General Hospital, Edinburgh, UK.

David Gilmour, Gartnavel Hospital, NHS Greater Glasgow and Clyde, Great Western Rd, Glasgow G12 0YN, UK

Andreea Ionean, Aberdeen Royal Infirmary, NHS Grampian, Forresterhill Road, Aberdeen, AB25 2ZN, UK

Nick George, Ninewells Hospital, NHS Tayside, James Arrott Dr, Dundee, DD2 1SG, UK

Adrian Dockery, Mater Misericordiae University Hospital, Dublin, Ireland.

Laura Finnegan, G. Jane Farrar, School of Genetics & Microbiology, Trinity College Dublin, Ireland.

## Abbreviations

IRD: Inherited Retinal Disease
RPE65: Retinal Pigment Epithelium-Specific 65 kDa
AR: Autosomal Recessive
WT: Wild Type
cDNA: Complementary DNA
AD: Autosomal Dominant
BCVA: Best-Corrected Visual Acuity
OCT: Optical Coherence Tomography
SW-AF: Short-Wavelength Autofluorescence
srWGS: Short-Read Whole Genome Sequencing
PCR: Polymerase Chain Reaction
bp: Base Pairs
SNVs: Single Nucleotide Variants
SVs: Structural Variants
WES: Whole Exome Sequencing
CNVs: Copy Number Variations
lrWGS: Long-Read Whole Genome Sequencing
ONT: Oxford Nanopore Technologies
h: hours
NEB: New England Biolabs
FBS: Fetal Bovine Serum
CETSA: Cellular Thermal Shift Assay
Co-IP: Co-immunoprecipitation
VUS: Variant of Unknown Significance
UK: United Kingdom
IQR: InterQuartile Range
Hypo-AF: Hypo-AutoFluorescent
Hyper-AF: Hyper-AutoFluorescent
EZ: Ellipsoid Zone
RCD: Rod-Cone Dystrophy
LOF: Loss Of Function
iPSC-RPE: induced Pluripotent Stem Cell-derived Retinal Pigment Epithelium
AMD: Age-Related Macular Degeneration
ASOs: Antisense Oligonucleotide
